# Chromosome instabilities in resynthesized *Brassica napus* revealed by FISH

**DOI:** 10.1007/s13353-020-00557-5

**Published:** 2020-04-22

**Authors:** Katarzyna Sosnowska, Maciej Majka, Joanna Majka, Jan Bocianowski, Marta Kasprowicz, Tomasz Książczyk, Laurencja Szała, Teresa Cegielska-Taras

**Affiliations:** 1 Department of Genetics and Breeding of Oilseed Crops, Plant Breeding and Acclimatization Institute - National Research Institute, Strzeszyńska 36, 60-479 Poznań, Poland; 2 Department of Genomics, Institute of Plant Genetics of the Polish Academy of Sciences, Strzeszyńska 34, 60-479 Poznań, Poland; 3 Department of Environmental Stress Biology, Institute of Plant Genetics of the Polish Academy of Sciences, Strzeszyńska 34, 60-479 Poznań, Poland; 4 Department of Mathematical and Statistical Methods, Poznań University of Life Sciences, Poznań, Poland; 5 Department of Pathogen Genetics and Plant Resistance, Institute of Plant Genetics of the Polish Academy of Sciences, Strzeszyńska 34, 60-479 Poznań, Poland

**Keywords:** Allopolyploids, Resynthesis, *Brassica napus*, Chromosome rearrangements, FISH, rDNA

## Abstract

*Brassica napus* is an allopolyploid plant, derived from spontaneous hybridization between *Brassica rapa* and *Brassica oleracea*. Intensive breeding has led to a significant reduction in genetic and phenotypic diversity within this species. Newly resynthesized hybrids from progenitor species may restore some diversity in *B. napus*, but they often are chromosomally and phenotypically unstable. Using fluorescence in situ hybridization, we tested chromosome constitutions in a range of new allopolyploids resynthesized from various parental species. A majority of these allopolyploids were euploid, with the expected chromosome numbers and constitutions, but deviations were also identified. We detected a low level of intergenomic rearrangements in analyzed hybrids and a high level of changes in rDNA loci. Our study revealed a significant effect of maternal cross combination on loss of 35S rDNA loci, especially when *B. rapa* was the maternal parent. The studied lines were characterized by diversified of pollen viability. In the analyzed hybrids, the erucic acid level in the seed oil ranged from 0 to 43.4% and total glucosinolate content in seeds ranged from 24.3 to 119.2 μmol g^−1^. Our study shows that cytogenetic analysis of *B. napus* resynthesized hybrids would be useful in breeding for the selection of lines with important agricultural characters and genetically stable stock seed production.

**Electronic supplementary material:**

The online version of this article (10.1007/s13353-020-00557-5) contains supplementary material, which is available to authorized users.

## Introduction

Polyploidy is a common phenomenon in flowering plant evolution, leading to biodiversity and the rapid formation of new species (Wood et al. 2009; Madlung 2013). Many polyploid plants are allopolyploids that have evolved from interspecific crosses of two or more species, either through the fusion of unreduced gametes or through interspecific hybridization followed by spontaneous chromosome doubling (Parisod et al. 2010; Soltis et al. 2010; Pires and Gaeta 2011; Malek et al. 2012). Interspecific hybridization between different species and distinct genomes can cause extensive genetic instability, e.g., genome rearrangements (Song et al. 1995; Pontes et al. 2004; Udall et al. 2005), parental DNA sequence elimination (Han et al. 2005), transposon activation, transposon-induced insertional mutagenesis (Kashkush et al. 2003; Madlung et al. 2005), gene conversion (Kovarik et al. 2004, 2005), epigenetic changes (Adams et al. 2003; Levy and Feldman 2004; Książczyk et al. 2011), and a wide range of further structural or functional genome modifications (Gaeta et al. 2007; Szadkowski et al. 2010; Zou et al. 2010; Pires and Gaeta 2011; Majka et al. 2018).

*Brassica napus* (2n = 4x = 38, genome composition AACC) is an example of an allotetraploid plant, derived from spontaneous crosses between two diploid genomes comprising the A-genome progenitor (*B. rapa*, 2n = 2x = 20) and the C-genome progenitor (*B. oleracea*, 2n = 2x = 18) (U 1935; Parkin et al. 1995; Hasterok and Maluszynska 2000). Exchanges between homoeologous chromosomes are extensive in this species and have the effect of creating novel allele combinations and phenotypic variation in newly formed *B. napus* allopolyploids (Osborn et al. 2003; Udall et al. 2005; Gaeta et al. 2007; Ge et al. 2009). Fujii and Ohmido (2011) reported that the consequences of aberrant meiosis may result in abnormal chromosome number and structure, which can lead to atypical phenotypes.

Cytogenetic characterization of *Brassica* karyotypes by fluorescence in situ hybridization (FISH) with molecular probes (i.e., 5S and 35S rDNA, BAC clones) provides valuable information on the structure, composition, and organization of genomes and chromosomes, as well as being helpful in establishing the taxonomy of species (Maluszynska and Heslop-Harrison 1993; Kulak et al. 2002; Książczyk et al. 2011; Xiong and Pires 2011; Catalan et al. 2012). Maluszynska and Heslop-Harrison (1993) carried out physical mapping of rDNA sequences using FISH in six *Brassica* species (*B. campestris* (syn. *B. rapa*), *B. carinata*, *B. juncea*, *B. napus*, *B. nigra*, and *B. oleracea*) and revealed large variability in the number of rDNA loci, ranging between two and five per haploid set. Using the same set of probes, Kulak et al. (2002) identified eight out of nineteen pairs of chromosomes in *B. napus*, ten out of eighteen pairs in *B. juncea*, and six out of sixteen pairs in *B. carinata*. Moreover, Xiong et al. (2011), using 45S, 5S, CentBr1, CentBr2, and BAC clones containing repetitive sequences, identified all of the homoeologous chromosomes in resynthesized (RS) *B. napus* allopolyploids*.* Karyotype analysis of RS plants from the S_10:11_ generation revealed aneuploidy, inter- and intragenomic rearrangements, chromosome breakage and fusion, and rDNA changes, as well as loss of repeat sequences. A new nomenclature system that follows the international linkage group system for *Brassica* was presented by Xiong and Pires (2011), and they established robust karyotypes of *B. rapa*, *B. oleracea*, and *B. napus* using FISH with rDNA, CentBr, and BAC clones as probes.

The FISH technique has proved to be useful for recognition of chromosomal aberrations, identification of chromosomes, and changes at rDNA loci, as well as intergenomic translocations in hybrids (Fukui et al. 1998; Maluszynska and Hasterok 2005; Xiong et al. 2011; Pellicer et al. 2013; Majka et al. 2017). DNA sequences encoding the 35S (18S-5.8S-26S) and 5S ribosomal RNA genes are organized in tandem arrays at one or more chromosomal loci, and their characteristic positions provide useful markers for chromosome identification and consequently detection of chromosome variability (Hasterok et al. 2001, 2006; Snowdon et al. 2002; Książczyk et al. 2011; Sochorová et al. 2017). The BoB014O06 BAC clone, derived from the *B. oleracea* BAC (BoB) library, hybridizes specifically to all C-genome chromosomes and allows the visualization of the C-genome for each *B. napus* genotype (Leflon et al. 2006; Książczyk et al. 2011; Ohmido et al. 2015). Using rDNA-FISH mapping combined with the C-genome-specific BAC probe, it is possible to recognize chromosome pairs: A1 (bearing 5S and 35S rDNA in opposite arms), A3 (bearing 35S rDNA (NOR) and 5S rDNA), A10 and C4 (bearing 5S rDNA), C7 (35S rDNA) and C8 (35S rDNA (NOR)) (Hasterok et al. 2006; Xiong and Pires 2011). Additionally, this approach can reveal chromosome rearrangements between the A and C genomes in RS lines of *Brassica* (Szadkowski et al. 2010; Książczyk et al. 2011; Xiong et al. 2011; Niemann et al. 2017). Many studies have demonstrated genetic changes caused by homoeologous recombination between ancestral genomes (Parkin et al. 1995; Udall et al. 2005; Xiong et al. 2011; Niemann et al. 2017), which can contribute to the formation of new gene combinations and can also destabilize the karyotype (Gaeta and Pires 2010). Thus, in studying hybrid forms, it is important to verify their genetic variation, which might be detected at the chromosome level.

Populations of natural and RS *B. napus* exhibit chromosome instability and recombination, which changes the arrangement of rDNA loci and results in locus gain or loss (Hasterok et al. 2001, 2006; Niemann et al. 2017). Maluszynska and Heslop-Harrison (1993) revealed that allopolyploid *B. napus* genotypes have fewer rDNA loci than the sum of the loci of their diploid ancestors; other studies confirmed this observation (Hasterok et al. 2001; Xiong et al. 2011). The A genome from *B. rapa*, as reported in the literature, is characterized by a higher frequency of changes in marker chromosomes, compared with those observed in the C genome from *B. oleracea* (Xiong et al. 2011; Sochorová et al. 2017).

The rapid development of oilseed rape cultivation in the world is mainly due to the marked progress in research and breeding programs of this plant. The increasing interest in oilseed rape is attributable to the extensive use of oilseed rape for the production of edible oil and feed ingredients, as well as for technological purposes. The currently cultivated cultivars of *B. napus*, so-called double-low (00) or canola types, clearly differ (in terms of the biochemical composition of seed) from traditional cultivars (Friedt et al. 2007). Erucic acid has been eliminated from seed oil content (in traditional cultivars, it represents 48–54% seed oil, while in “00” cultivars it comprises ≤ 2%), and the glucosinolate content in seeds has been significantly reduced (in traditional cultivars, its content is 110–160 μmol g^−1^ of seed and in “00” cultivars it is 8–15 μmol g^−1^ of seed). All current *B. napus* cultivars have genes determining the lack of erucic acid; these derive from the zero erucic cultivar ‘Liho’ (Stefansson et al. 1961). Genes governing low glucosinolate content derive from the spring cultivar of oilseed rape ‘Bronowski’ (Krzymański 1968).

Together, these genetic characteristics significantly reduce the genetic and phenotypic diversity of *B. napus*. Therefore, in collections of the genus *Brassica*, there is a need for new sources of variation. One way to expand the genetic resources for breeding oilseed rape is to obtain artificial RS allopolyploids by crossing the progenitor species *B. oleracea* and *B. rapa*. Resynthesized oilseed rape was obtained as a result of crosses between *B. rapa* and *B. oleracea* using two methods: in vivo pollination (Sosnowska et al. 2010) and in vitro placental pollination (Sosnowska and Cegielska-Taras 2014) and then through in vitro culture of isolated embryos in the early stage of their development (embryo rescue culture). Molecular analysis using AFLP markers and 10 primer combinations performed in our previous studies showed a high degree of diversity among one hundred *Brassica* genotypes, including de novo synthesized oilseed rape lines (Sosnowska et al. 2017). Natural accessions of *B. napus* generate a compact group, which is distinct from RS and semi-RS lines. Additionally, it was demonstrated that the RS plant group is characterized by greater genetic variation than the cultivated oilseed rape group. The same RS lines were cytogenetically analyzed in this work.

The main aim of this study was to determine the number and structure of chromosomes and to track possible chromosomal rearrangements in RS allopolyploids of *B. napus* obtained by crossing various genotypes of parental species of *B. napus*. Additionally, the variability of selected phenotypic traits in RS lines was evaluated.

## Materials and methods

### Plant material

The following species were used in this study: 32 lines of resynthesized (RS) *B. napus* allopolyploids and their diploid progenitor species; four genotypes of *B. oleracea*; and eight genotypes of *B. rapa* (Table 1). Cultivars of *B. rapa* were selected based on their quality (“00”: ‘Premium’, ‘Salut’, ‘BOH 2877’; “0+”: ‘Kova’ and ‘Skye’; “++”: ‘Ludowy’), growth habit (spring and winter; Table 1), and other traits (pak choy exhibits high tolerance to low temperatures and Chinese cabbage ‘Kilakin’ is resistant to clubroot). Curly kale is characterized by high winter hardiness and low soil requirements, and is resistant to several diseases, while brussels sprouts cv. ‘Crispus’ is also a source of resistance to clubroot. Seeds of parental materials were obtained from Plant Breeding Strzelce Ltd., Co., Poland, and commercial sources, while synthetic allotetraploid lines were obtained by crossing *B. rapa* and *B. oleracea* as maternal and paternal genome donors, respectively. Young S_0_ plants were treated with colchicine for chromosome doubling. (Sosnowska and Cegielska-Taras 2014). We had 32 S_0_ plants which represented 12 genotype combinations, including three genotype combinations involved in both reciprocal cross (*B. rapa* × *B. oleracea* and *B. oleracea* × *B. rapa*), and we planted one randomly selected seed for each subsequent generation up until the S_3_ generation. During flowering, plants were self-pollinated to generate S_1_ seeds and then to generate S_2_ and S_3_ seeds (Fig. S1). In S_0_ generation-10 lines; in S_1_ generation-5 lines (at least 3 plants from each line); in S_2_ generation-15 lines (at least 3 plants from each line); and in S_3_ generation-2 lines (at least 3 plants from each line) were cytogenetically analyzed. Seeds were germinated on filter paper moistened with tap water at room temperature (22 ± 1 °C) in the dark until the roots were 1.5 to 2.0 cm long. For RS lines analyzed in S_0_ generation only, roots were taken directly from the plants when they were identified as sterile. Consequently these lines were lost. To ensure suitable condensation of chromosomes at metaphase, whole seedlings were treated with 2 mM 8-hydroxyquinoline for 3 h at room temperature, followed by fixation in a 3:1 (*v/v*) ethanol:glacial acetic acid mixture, and then stored at − 20 °C until use.

**Table 1:**
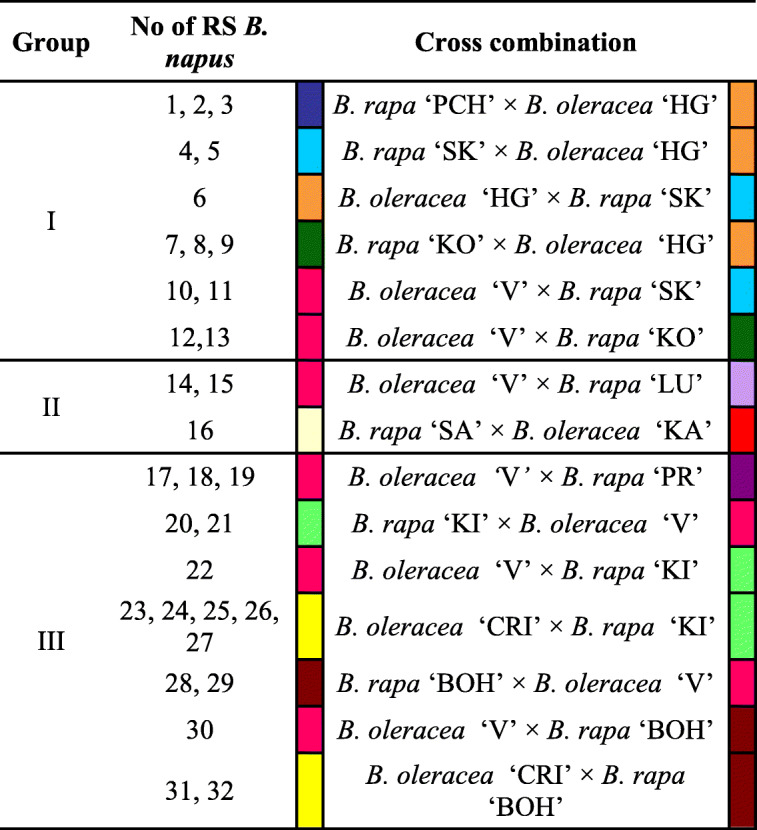
The origin of RS *B. napus* plants obtained from reciprocal crosses of different *B. rapa* and *B. oleracea* parental forms. The colors in the table are assigned to the particular parental forms, used in interspecific hybridization*. B. oleracea*: ssp. *acephala* var. *sabellica* (curly kale) cvs:‘Vitessa’ (V), ‘Halbhoher Grüner’ (HG), ‘Kapral’ (KA); ssp. *fruticosa* var. *gemmifera* (brussels) cv. ‘Crispus’(CRI) and *B. rapa*: ssp. *rapifera* (turnip rape), spring type: cvs. ‘Kova’ (KO) and ‘Skye’ (SK) and winter type: cvs. ‘Ludowy’ (LU), ‘Premium’ (PR), ‘Salut’ (SA) and ‘BOH 2877’ line (CR); ssp. *chinensis* var. *chinensis* (pak choy; PCH) and ssp. *pekinensis* (Chinese cabbage) cv. ‘Kilakin’ (KI). Three groups of RS *B. napus* plants (I, II, and III) based on three various A-genome like rDNA loci patterns which were found in their parental forms (A1/A3/A5/6/9/A10: I—4/2/4/2; II—2/2/2/2; III—2/2/6/2); C-genome like rDNA loci pattern was constant among the parental forms

### Chromosome preparations

Meristematic cells of root tips were used as a source of mitoses. Fixed seedlings were washed in 0.01 M citric acid/sodium citrate buffer (pH 4.6–4.8) for 20 min prior to enzymatic digestion in a mixture comprising 20% (*v/v*) pectinase (Sigma), 1% (*w/v*) cellulase (Calbiochem), and 1% (*w/v*) cellulase “Onozuka R-10” (Serva) for 1.5–2.5 h at 37 °C. The root tips were squashed in a drop of 45% acetic acid. Cover slips were removed by freezing, and the preparations were post-fixed in chilled 3:1 (*v/v*) ethanol:glacial acetic acid mixture, followed by dehydration in absolute ethanol and air dried (Hasterok et al. 2001). Chromosome preparations made from at least three individual plants from each accession were subjected to FISH analysis. Chromosome analysis was carried out on 5–10 well-spread metaphases. Each chromosomal preparation was derived from a different single root tip, so that each preparation corresponded to one individual.

### DNA probes

The species-specific BoB014O06 BAC clone from the *B. oleracea* BAC library was used as a probe for the C-genome (Howell et al. 2002; Leflon et al. 2006; Książczyk et al. 2011; Niemann et al. 2017). The BoB014O06 BAC clone was labeled by random priming with digoxigenin-11-dUTP (Roche). The ribosomal probes used in this study were 26S rDNA (Unfried and Gruendler 1990), used for detection of 35S rDNA loci, and pTa794 (Gerlach and Dyer 1980), which contained the 5S rDNA. The labeling procedure followed the methods described in detail by Książczyk et al. (2011).

### Fluorescence in situ hybridization and chromosome identification

The FISH procedure was adapted as described by Hasterok et al. (2006) with minor modifications. The slides were pretreated with RNase (100 μg/ml) in 2× SSC at 37 °C for 1 h, washed in 2× SSC, and dehydrated in ethanol. The hybridization mixture consisted of 50% deionized formamide, 20% dextran sulfate, 2× SSC, salmon sperm blocking DNA in 75–100× excess of labeled probes, and 2.5–3.0 ng/μl of each DNA probe. In all experiments, the hybridization mixture was pre-denatured at 75 °C for 10 min and applied to the chromosome preparations. Slides and the pre-denatured hybridization mixture were then denatured together at 75 °C for 4.5 min and allowed to hybridize overnight in a humid chamber at 37 °C. After stringent washes (10% deionized formamide in 0.1× SSC at 42 °C, an equivalent of 79% stringency), the immunodetection of digoxigenin-labeled probes was carried out with anti-digoxigenin antibody conjugated with FITC (Roche). The preparations were mounted and counterstained in Vectashield (Vector Laboratories) containing 2.5 μg ml^−1^ of 4′,6-diamidino-2-phenylindole (DAPI) (Sigma). All images were acquired using an Olympus XM10 CCD camera attached to an Olympus BX 61 automatic epifluorescence microscope. Image processing and superimpositions were carried out using Olympus Cell-F imaging software and Micrografx Picture Publisher software.

The nomenclature of rDNA-bearing chromosomes used in our research was proposed by Xiong and Pires (2011), with additional chromosome numbering by Hasterok et al. (2001, 2006).

### Pollen viability

Pollen viability of RS plants was determined in mature anthers based on pollen staining in 1% acetocarmine using microscopic examination and then counting viable and non-viable pollen grains in 10 fields of view. Pollen grains were taken from randomly selected flowers. Round, intensely stained pollen grains were regarded as apparently fertile, while shrunken, deformed, and poorly stained grains were scored as sterile.

### Biochemical analysis

The fatty acid composition in the oil and the glucosinolate content in the seed were determined by gas chromatography (Michalski et al. 1995). Seed quality was determined for 20 RS lines (for which a suitable seed mass was collected).

### Statistical analysis

One-way analysis of variance was carried out to determine the effects of RS lines and cultivar of natural *B. napus* on the variability of pollen viability. The position and variability characteristics of studied genotypes, in terms of pollen fertility, were presented in the form of boxplot. Differences between values of A-genome chromosomes, C-genome chromosomes as well as gain and loss of rDNA loci for A × C and C × A crossing combinations were tested on the basis of *t* test. One-way analysis of variance (ANOVA) was carried out to determine the effects of genotypes on the variability of pollen viability. The Tukey’s honestly significant difference (HSD) was calculated for pollen viability, and on this basis homogeneous groups were determined. The relationship between erucic acid content and total glucosinolate content was estimated based on Pearson’s correlation coefficients. All the analyses were conducted using the GenStat v. 18 statistical software package.

## Results

### FISH analysis of resynthesized *B. napus* allopolyploids

In total, 32 lines of RS *B. napus* allopolyploids and their diploid progenitor species, comprising four genotypes of *B. oleracea* (2n = 2x = 18; C genome) and eight genotypes of *B. rapa* (2n = 2x = 20; A genome), were analyzed.

**Fig. 1:**
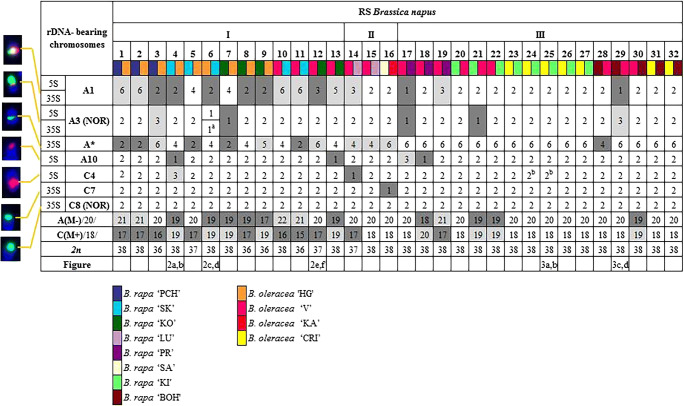
Number of rDNA sites in particular resynthesized *B. napus* plants as indicated by FISH with the 5S and 35S probes*.* The A and C typical rDNA-bearing chromosomes are shown at the left. Three groups of RS *B. napus* plants (I, II, and III) based on three various A-genome like rDNA loci patterns which were found in their parental forms (A1/A3/A5/6/9/A10: I—4/2/4/2; II—2/2/2/2; III—2/2/6/2); C-genome like rDNA loci pattern was constants among the parental forms. The detection of additional A- and C-genome like chromosomes is highlighted in light gray color; the loss of A- and C-genome like ones is highlighted in dark gray color. A(M-) A-genome like chromosomes (20) not marked by the C-genome specific sequence, C(M+) C-genome like chromosomes (18) marked by the C-genome-specific sequence. ^a^The A-genome NOR that showed extended signals of decondensed chromatin far beyond the chromosome arm. ^b^The C-genome like chromosome that showed the presence of a highly polymorphic additional site of 35S rDNA which is localized interstitially in the short arm. The colors in the figure are assigned to the particular parental forms, used in interspecific hybridization

The majority of the allopolyploids were tetraploid with 38 chromosomes (78% of all RS lines), whereas the remaining RS plants were hypoploids with either 2n = 36 or 37, constituting 13% and 9% of all RS lines, respectively (Fig. 1). Moreover, FISH experiments with the C-genome-specific BAC probe showed that 47% of allopolyploids were characterized by a different ratio of parental chromosomes, i.e., 18–22 A-genome chromosomes and 16–20 C-genome chromosomes. In the hypoploids, the number of A- and C-genome chromosomes was in the range of 17–21 and 15–19, respectively. We identified ten lines with a reduced number of A-genome chromosomes, nine lines with a reduced number of C-genome chromosomes and one line where the number of chromosomes of both genomes was lower than in the progenitors. Thirteen lines had the expected genome composition, i.e., 20 A-genome and 18 C-genome chromosomes (Fig. 1).

FISH mapping using a combination of the C-genome-specific BAC probe with rDNA probes revealed three groups of RS *B. napus* plants (I, II, and III) based on three distinct A-genome rDNA loci patterns found in their parental forms (A1/A3/A5/6/9/A10: I—4/2/4/2; II—2/2/2/2; III—2/2/6/2); the C-genome rDNA loci pattern was constant among the parental forms (Fig. S2). In each RS *B. napus* line studied, all changes to the expected number and position of rDNA sites in the A and C chromosomes were treated as possible variations in the rDNA loci pattern. By referring to the patterns of loci in the parental forms, it was apparent where addition or loss of A- and C-genome chromosomes had occurred in the RS lines (Fig. 1). We observed more changes in the number of rDNA loci of A-genome chromosomes, especially chromosome A1, than in the C-genome chromosomes, with no changes at all in chromosome C8. We identified eight lines from group III (which comprised 16 lines in total) with no changes in number of chromosomes or rDNA loci (Fig. 1). The results of FISH performed on the parental forms and their progeny, obtained by subsequent self-pollinations of RS *B. napus* (Table 1), revealed that the number of rDNA loci changes approximately doubled for each subsequent generation of RS plants. The mean frequency of rDNA loci changes per line was as follows: 0.4 for the S_0_ generation, 1 for S_1_, 2.9 for S_2_, and 4.5 for S_3_ (Fig. S2).

**Fig. 2:**
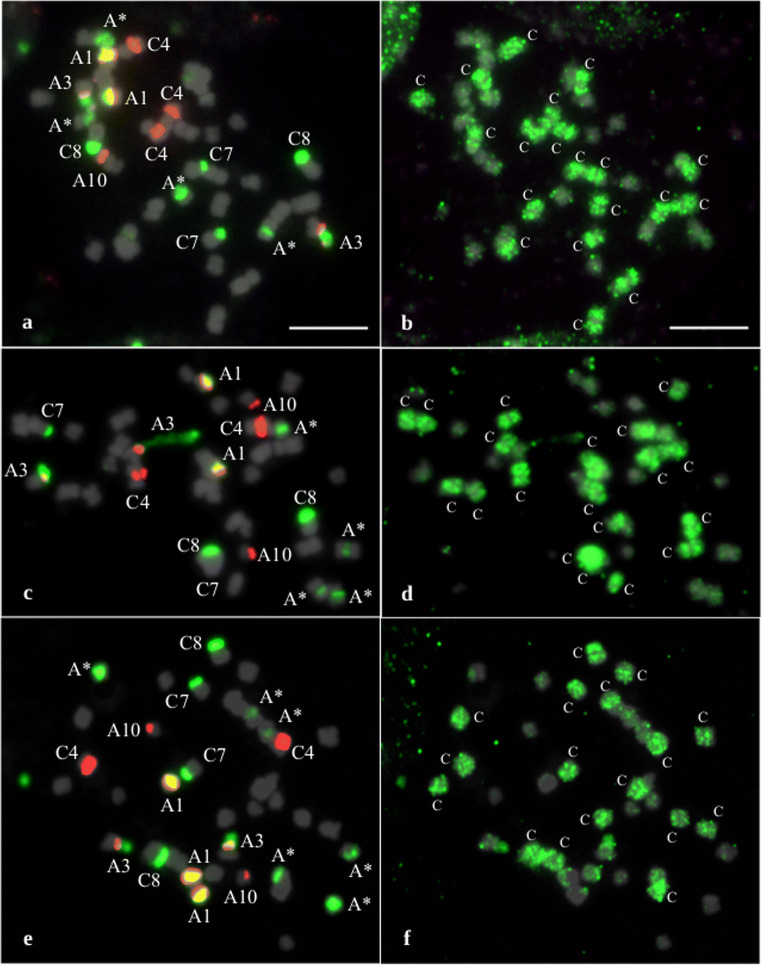
Chromosome identification of parental species in resynthesized *B. napus* allotetraploids using rDNA-FISH (a, c, e) and BAC-FISH (b, d, f). FISH images were created using probes as follows: (i) 5S rDNA (*red*), (ii) 35S rDNA (*green*), and (iii) BoB014O06 (*green*); chromosomes were counterstained with DAPI (*gray*). a, b RS4 plant [19A + 19C]. c, d RS6 plant [19A + 19C]. e-f RS12 plant [20A + 17C]. *Uppercase letters* denote the genomic origin of tagged chromosomes; *Arabic numerals* refer to the nomenclature of rDNA-bearing chromosomes; *Represents chromosomes A5, A6, and A9. *Scale bars* 5 μm

As an example of a line with an expected number of chromosomes (2n = 38) but aneuploid is the RS4 line. BAC-FISH analysis showed that this line had 19 A-genome and 19 C-genome chromosomes (instead of 20 A-genome and 18 C-genome chromosomes). Within A-genome, we observed loss of one chromosome A10 and an additional chromosome C4 in C-genome (Fig. 2a, b). Similarly, in the RS6 line, we identified the same chromosome ratio of both genomes and loss of two A1 chromosomes, but the A-genome NOR (chromosome A3) showed extended signals of decondensed chromatin far beyond the chromosome arm (Fig. 2c, d). FISH analysis of the RS12 line revealed the presence of 20 A-genome and 17 C-genome chromosomes along with the loss of one chromosome A1 (Fig. 2e, f).

**Fig. 3:**
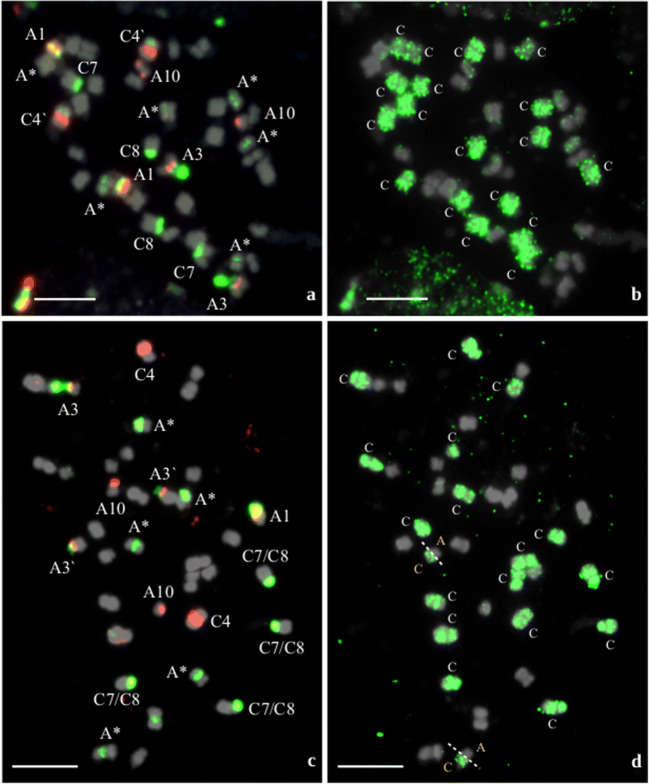
Chromosome identification of parental species in resynthesized *B. napus* allotetraploids using rDNA-FISH (a, c) and BAC-FISH (b, d). Chromosomes were hybridized simultaneously with the 5S (*red*), 35S (*green*), and BoB014O06 (*green*) probes; chromosomes were counterstained with DAPI (*gray*). a, b RS25 plant [20A + 18C]. c, d RS29 plant [20A + 18C]. *Uppercase letters* denote the genomic origin of tagged chromosomes; *Arabic numerals* refer to the nomenclature of rDNA-bearing chromosomes; *Represents chromosomes A5, A6, and A9; *white lines* with intervals indicating recombination breakpoints. *Scale bars* 5 μm

It is worth mentioning that in some of the RS lines with the predicted number of chromosomes (2n = 38, i.e., 20 A-genome chromosomes and 18 C-genome chromosomes), we also identified changes in the number of rDNA loci. In RS25, for example, we found a C-genome-like chromosome (chromosome C4) with a highly polymorphic additional 35S rDNA locus, which is localized interstitially in the short arm (Fig. 3a, b). Moreover, in RS29, two pairs of chromosomes of the A- and C-genomes had recombined together, but this rearrangement did not influence the final chromosome number in the hybrid plants. In this line we observed only one loss and one gain of rDNA loci in chromosomes A1 and A3, respectively (Fig. 3c, d; Fig. S2). Similarly, to line RS6, in both RS25 and RS29, decondensation of 35S rDNA in the chromosomes A3 was also apparent.

We observed statistically significant difference only for loss of rDNA loci between A × C and C × A crossing combinations (*t* = 4.33, *p* < 0.001). More losses of rDNA loci occurred when *B. rapa* is the maternal genome donor and *B. oleracea* the paternal genome donor than in the opposite direction. Differences between values of A-genome chromosomes, C-genome chromosomes and gain in rDNA loci for A × C and C × A were not significant (for A: *t* = − 0.82, *p* = 0.419; for C: *t* = − 0.12, *p* = 0.902; and for gain in rDNA loci: *t* = 0.83, *p* = 0.411).

### Pollen viability

**Fig. 4:**
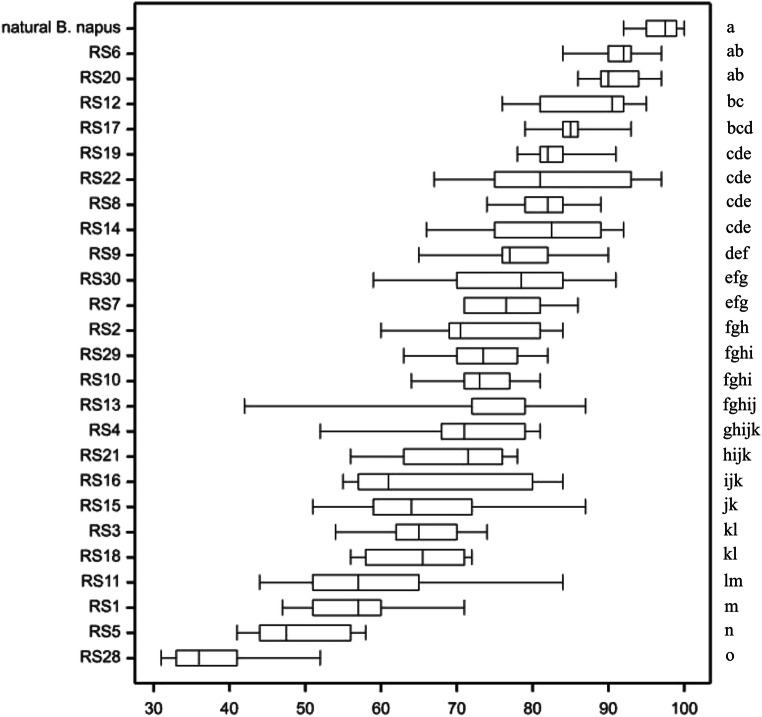
Pollen viability of RS lines and cultivar of natural *B. napus* (HSD_0.05_ = 6.881); genotypes marked with the same letter are not statistically different

Results of analysis of variance indicated that the effects of RS lines were significant for pollen viability. RS lines usually showed high variability of pollen viability, while the control plant (a cultivar of natural *B. napus*) was characterized by the lowest pollen variability and the highest viability of pollen (Fig. 4). Among the RS genotypes, the RS6 and RS20 lines were characterized by the highest viability of pollen grains. These two RS lines were not statistically different from natural *B. napus*. The lowest pollen viability was found in lines RS5 and RS28, where the *B. rapa* component was used as the maternal parent in the interspecific hybridization. In several lines, the viability of pollen grains was not studied because flowers of these lines had reduced anthers. The direction of crossing and the total number of chromosomes did not significantly affect the pollen viability of the analyzed RS lines.

### Biochemical analysis

**Fig. 5:**
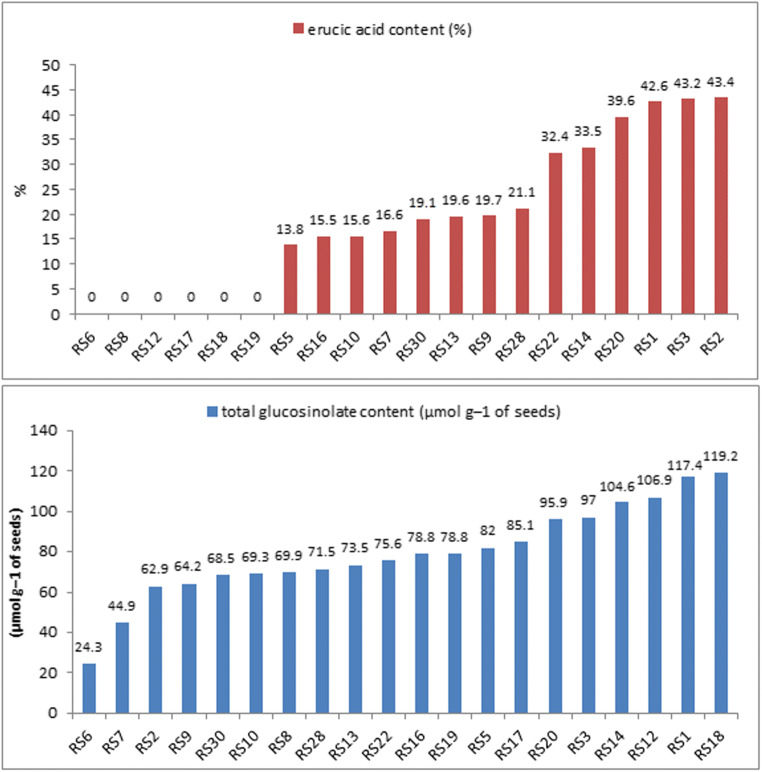
Erucic acid in oil and glucosinolates in seeds of RS lines

Fatty acid composition in oil and glucosinolate content in meal were measured in the seeds of RS lines. No seeds were collected for lines RS23, RS24, RS25, RS26, RS27, RS31, and RS32 (the flowers of these lines had reduced anthers), and not enough seeds were available for the RS4, RS11, RS15, RS21, and RS29 lines. Many of the RS lines produced seeds with high levels of erucic acid and glucosinolates. Only six RS lines (RS6, RS8, RS12, RS17, RS18, and RS19) possessed seeds with zero erucic acid content, but these same lines had a high level of glucosinolates (Fig. 5). The erucic acid level in the seed oil ranged from 0 to 43.4% and total glucosinolate content in seeds ranged from 24.3 to 119.2 μmol g^−1^. The calculated coefficients of variation for erucic acid content were 82.29% and for glucosinolates 28.39% (Table 2). The erucic acid content and total glucosinolate content were not statistically significant correlated (*r* = 0.215, *p* = 0.363).

**Table 2:** Statistical analysis of seed quality in the seed of 20 RS lines

Seed quality	Minimum	Maximum	Mean	Standard deviation	Coefficient of variation (%)
Erucic acid content (%)	0	43.4	18.79	15.46	82.29
Total glucosinolate content (μmol g^−1^ of seeds)	24.3	119.2	79.52	22.58	28.39

## Discussion

The cultivation and selection of *B. napus* has had a strong impact on its current limited diversity (Girke et al. 2012). In this paper, we report on the resynthesis of *B. napus* by sourcing A and C genomes from various genotypes of *B. rapa* and *B. oleracea*, respectively. Our crossing procedures included hybridization in both directions (*B. rapa* (♀) × *B. oleracea* (♂) and *B. oleracea* (♀) × *B. rapa* (♂)). The majority of the 32 RS lines had the correct allotetraploid genotype with 20 chromosomes derived from *B. rapa* and 18 chromosomes from *B. oleracea*. Nonetheless, lines with a different number of parental chromosomes were also observed (Fig. 1). It is known that an unbalanced number of donor genomes and aneuploidy have a destabilizing impact on meiotic cell division (Cai and Xu 2007; Zamariola et al. 2014). However, despite an unbalanced chromosome number and aneuploidy in some resynthesized *B. napus* lines, we observed only one case of chromosome interaction and rearrangement between A and C genomes (Fig. 3c, d). Homoeologous pairing between A and C parental genomes in newly RS *B. napus* plants was described by Szadkowski et al. (2010), who suggested that the first meiosis generates rearrangements in both genomes and promotes subsequent restructuring in further generations.

In our plant material, we identified only limited intergenomic rearrangement, as shown by FISH with a BAC clone specific for the C genome, but we report marked changes in rDNA loci. For 35S rDNA, we observed signals from 11 to 16 loci and for 5S rDNA from 7 to 12 loci. In natural accessions of *B. napus*, there are usually between 10 and 14 - 35S rDNA sites (Maluszynska and Heslop-Harrison 1993; Hasterok et al. 2006), but in synthetic lines Książczyk et al. (2011) reported 12 to 16 such loci. In our RS hybrids, the most frequent pattern constituted 14 - 35S rDNA signals (10 derived from *B. rapa* and 4 from *B. oleracea*) and 8 - 5S rDNA signals (6 derived from *B. rapa* and 2 from *B. oleracea*). Książczyk et al. (2011) also observed 14 - 35S rDNA loci and 8 - 5S rDNA signals in the S_0_ generation of *B. napus* hybrids. However, in the next generations (S_1_-S_3_) the variation in rDNA sites increased. Due to the limited resolution of FISH-based karyotyping for chromosomal rearrangements in *Brassica*, we may have missed smaller chromosome rearrangements. However, this approach has been used effectively in previous studies to detect chromosome rearrangements in synthetic *B. napus* lines (Książczyk et al. 2011; Niemann et al. 2017; Xiong et al. 2011).

In allopolyploid plants, especially in early generations of hybrids, rDNA sites may be affected by structural alterations in the chromosomes (Volkov et al. 1999; Xiong et al. 2011). Indeed, Książczyk et al. (2011) hypothesized that major chromosome instability can induce NOR restructuring. Moreover, in hybrid plants, although in principle all 35S rDNA loci can be active, some loci can be silenced as a result of nucleolar dominance (Navashin 1934; Pikaard 2000; Tucker et al. 2010). Among our plant lines with a changed number of parental chromosomes (i.e., different to 20 A-genome chromosomes and 18 C-genome chromosomes), we identified more variation in 35S rDNA loci compared with lines with the expected ratio of A- and C-genome chromosomes. This confirms that the marked instability in the number of parental chromosomes in synthetic hybrids is associated with variability in the number of 35S rDNA loci.

Taking into consideration the rDNA-bearing chromosomes of both genomes, the greatest variation was observed for chromosomes derived from the A genome. In genome A, it is possible to unequivocally recognize chromosome pairs by rDNA-FISH mapping: A1 (bearing 5S and 35S rDNA in opposite arms), A3 (bearing 35S rDNA (NOR) and 5S rDNA), and A10 (bearing 5S rDNA) (Hasterok et al. 2006; Xiong and Pires 2011). In our RS lines, we found that, within the A genome, the most variable were chromosomes A1, whose number ranged from 1 to 6 (Fig. 1). For chromosomes A3, we also observed changes in their number (ranged from 1 to 3), but the majority of RS plants possessed two such chromosomes in the karyotype. Intriguingly, Książczyk et al. (2011) observed decondensed 35S rDNA signals in the A3 chromosomes. They reported extended NOR fibers in all four identified A3 chromosomes of a RS line of *B. napus*. We also identified extended NORs in selected RS lines, but in particular lines we observed different numbers of decondensed signals, i.e., in one out of two chromosomes A3 (Fig. 2c), or one out of three chromosomes A3 (Fig. 3c) or two out of two chromosomes A3 (Fig. 3a). This phenomenon is most likely related to the activation and inactivation of rRNA genes. Neves et al. (2005) showed that, during metaphase, condensed and decondensed chromatin was intermingled at NOR sites. Interestingly, we found extended NORs derived from the A genome, while 35S rDNA sites in chromosomes C8 (NORs derived from *B. oleracea* genome) were highly condensed (Figs. 2 and 3). Moreover, the number of NORs in C8 chromosomes was stable in all the plants we analyzed. We hypothesize that these NOR regions are transcriptionally inactive, which might explain why their number does not vary in hybrid plants.

The most commonly lost in A genome loci were observed for A1 and A3 chromosomes, whereas C7 and C8 were relatively untouched, what is in relevance with the known homoeologous recombination frequencies previously elucidated—C8 rarely recombine with its A-genome homoeologs (A8 and A9), and C7 even more rarely with its homoeologous partners, but A1-C1 is the most common homoeologous pair, and A3-C3 recombinants are also more frequent (Nicolas et al. 2007, 2009; Udall et al. 2005). Our study revealed a significant effect of maternal cross combination on loss of 35S rDNA loci, especially when *B. rapa* was the maternal parent. That is in relevance with the previous reports that A-genome is characterized by higher frequency of changes in marker chromosomes, compared with those observed in the C genome from *B. oleracea* (Fig. S2). A possible confounding factor in testing for the effect of genotype is that parent *B. rapa* and *B. oleracea* genotypes were heterozygous. Hence, any genetic factors which may promote or prevent loss or gain of 35S rDNA loci may also be segregating between individual RS lines, despite these lines being of the same genotype composition. However, we would still expect lines within a single genotype combination to be more similar to each other than to lines from different genotype combinations.

Chromosomal rearrangements in *B. napus* caused by homoeologous recombination are widespread and have effects on phenotypic diversity that can lead to aberrant meiotic behavior and consequently reduced fertility (Ge et al. 2009; Gaeta and Pires 2010; Fujii and Ohmido 2011). The pollen viability of the studied lines was varied (Fig. 4). The plants with 38 chromosomes did not always show high pollen fertility. Fujii and Ohmido (2011) reported that RS plants with low pollen fertility contain aberrant chromosome fragments in somatic cells. Evaluation of pollen viability is an important characteristic of the genotype because the ability to pollinate is one of the basic selection features in plant breeding processes (Lankinen et al. 2018). RS plants originating from interspecific crosses between *B. rapa* and *B. oleracea* are usually self-incompatible, a feature transferred from the parental species. The unstable meiotic behavior of the parental genomes when they are brought together in RS lines results in low seed set capability (Rahman 2005). Analysis of pollen viability may indicate the cause of the difficulties in self-pollinated plants. However, hand-assisted pollination of closed floral buds of a hybrid plant by its own pollen allows at least a few seeds to be obtained.

The seed quality parameters of hybrid plants depend on which parental forms are used. There are no reports on double-low cultivars of *B. oleracea* apart from zero erucic acid cabbage mutant ‘Kashirka 202’ (Rygulla et al. 2007). The majority of our RS genotypes display low seed quality traits (Table 2). Seyis et al. (2005) revealed that crosses of genotypes with low erucic acid content usually result in zero erucic acid RS *B. napus*. We confirmed this using double-low or zero erucic acid cultivars of *B. rapa*. These RS allopolyploids require improvement before being introduced to breeding practice (Friedt et al. 2003; Chatterjee et al. 2016; Szała et al. 2016).

Cytogenetic analysis of the RS *B. napus* lines enabled the number and structure of chromosomes to be determined and chromosomal rearrangements to be tracked. Such lines, created as a result of interspecific hybridization, would be useful in breeding for the selection of lines with important agricultural characters and genetically stable stock seed production. Our study provides a valuable resource for further genetic investigations of genome organization and genomic stability of novel RS *B. napus* genotypes.

## Electronic supplementary material

ESM 1 (PDF 247 kb)

ESM 2 (PDF 674 kb)

## Abbreviations

BAC: Bacterial artificial chromosome
FISH: Fluorescence in situ hybridization
NOR: Nucleolus organizer region
RS: Resynthesized
00-quality: Genotypes with zero erucic acid and low seed glucosinolate content
0+ quality: Genotypes with zero erucic acid and high glucosinolate content
++ quality: Genotypes with high erucic acid and high glucosinolate content
