# Supplementary material for: Chromosome instabilities in resynthesized Brassica napus revealed by FISH
Source: J Appl Genet. 2020 Apr 22;61(3):323–35. doi: 10.1007/s13353-020-00557-5 (PMC7413880; doi:10.1007/s13353-020-00557-5)

Fig. S1 Scheme showing the development of S_0, S_1, S_2, S_3 generations of RS *Brassica napus* lines. One randomly selected seed for each subsequent generation was used up until the S_3 generation

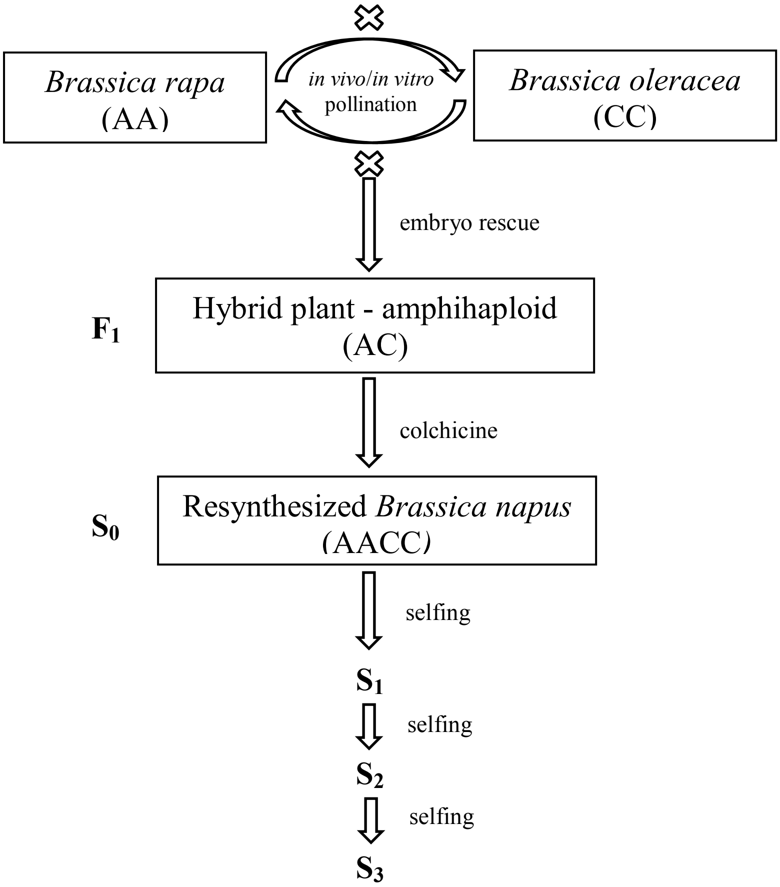

Supplement: Supplementary file 1 — (PDF 247 kb) [file 13353_2020_557_MOESM1_ESM.pdf]
