# Supplementary material for: Chromosome instabilities in resynthesized Brassica napus revealed by FISH
Source: J Appl Genet. 2020 Apr 22;61(3):323–35. doi: 10.1007/s13353-020-00557-5 (PMC7413880; doi:10.1007/s13353-020-00557-5)
Supplement: Supplementary file 2 — (PDF 674 kb) [file 13353_2020_557_MOESM2_ESM.pdf]

Fig. S2 Number of rDNA sites in diploid parental forms and their resynthesized allotetraploids. Three groups of RS *B. napus* plants (I, II and III) based on three various A-genome like rDNA loci patterns which were found in their parental forms (A1/A3/A5/6/9/A10: I – 4/2/4/2; II – 2/2/2/2; III – 2/2/6/2); C-genome like rDNA loci pattern was constant among the parental forms. The detection of additional A- and C-genome like chromosomes is highlighted in *green*, the loss of A- and C-genome like ones is highlighted in *red*. m- means A-genome like chromosomes (20) not marked by the C-genome specific sequence, m+ means C-genome like chromosomes (18) marked by the C-genome-specific sequence; n.a. – not analyzed. * cytogenetically undistinguishable A5, A6, and A9 chromosomes; ** The base chromosomal types, numbered I–VIII, have been introduced and described in detail by Hasterok et al. (2001), with the exception that the 5S rDNA site in chromosome type V is now assigned to the short arm (Hasterok et al. 2006). *** C genome-specific BAC-based probe

			S_2-S_3 generation					S_1-S_2 generation				S_2 generation			S_1-S_2 generation					S_2 generation			
**rDNA marker****			**PCH**	**HG**	**RS1**	**RS2**	**RS3**	**SK**	**HG**	**RS4**	**RS5**	**HG**	**SK**	**RS6**	**KO**	**HG**	**RS7**	**RS8**	**RS9**	**V**	**SK**	**RS10**	**RS11**
1	2	*2n*	20	18	38	38	36	20	18	38	37	18	20	38	20	18	38	36	36	18	20	38	36
**A-genome**																							
A3	I		**2**		2	2	3	**2**		2	2		**2**	1+1	**2**		1	2	2		**2**	2	2
A1	II		**4**		6	6	2	**4**		2	4		**4**	2	**4**		4	2	2		**4**	6	6
A10	V		**2**		2	2	2	**2**		1	2		**2**	2	**2**		2	2	2		**2**	2	2
A5/6/9*	VIII		**4**		2	2	6	**4**		4	2		**4**	4	**4**		2	4	5		**4**	4	2
**C-genome**																							
C4	IV			**2**	2	2	2		**2**	3	2	**2**		2		**2**	2	2	2	**2**		2	2
C8	VI			**2**	2	2	2		**2**	2	2	**2**		2		**2**	2	2	2	**2**		2	2
C7	VII			**2**	2	2	2		**2**	2	2	**2**		2		**2**	2	2	2	**2**		2	2
**BAC marker*****																							
A-genome (m-)			n.a.	n.a.	21	21	20	n.a	n.a	19	20	n.a	n.a.	19	n.a	n.a.	19	19	17	n.a.	n.a.	22	21
C-genome (m+)					17	17	16			19	17			19			19	17	19			16	15

		S_2 generation				S_2 generation				S_2 generation			S_2 generation					S_1 generation			
**rDNA marker****		**V**	**KO**	**RS12**	**RS13**	**V**	**LU**	**RS14**	**RS15**	**SA**	**KA**	**RS16**	**V**	**PR**	**RS17**	**RS18**	**RS19**	**KI**	**V**	**RS20**	**RS21**
1	2	18	20	37	38	18	20	37	38	20	18	38	18	20	38	38	38	20	18	38	38
**A-genome**																					
A3	I		**2**	2	2		**2**	2	2	**2**		2		**2**	1	2	2	**2**		2	1
A1	II		**4**	3	5		**2**	3	2	**2**		2		**2**	1	2	3	**2**		2	2
A10	V		**2**	2	1		**2**	2	2	**2**		2		**2**	3	1	2	**2**		2	2
A5/6/9*	VIII		**4**	6	4		**2**	4	4	**2**		6		**6**	6	6	6	**6**		6	6
**C-genome**																					
C4	IV	**2**		2	2	**2**		1	2		**2**	2	**2**		2	2	2		**2**	2	2
C8	VI	**2**		2	2	**2**		2	2		**2**	2	**2**		2	2	2		**2**	2	2
C7	VII	**2**		2	2	**2**		2	2		**2**	1	**2**		2	2	2		**2**	2	2
**BAC marker*****																					
A-genome (m-)		n.a.	n.a.	20	19	n.a.	n.a.	20	20	n.a.	n.a.	20	n.a.	n.a.	20	18	21	n.a.	n.a.	20	19
C-genome (m+)				17	19			17	18			18			18	20	17			18	19

		S_1 generation			S_0 generation							S_0 generation				S_0 generation			S_0 generation			
**rDNA marker****		**V**	**KI**	**RS22**	**CRI**	**KI**	**RS23**	**RS24**	**RS25**	**RS26**	**RS27**	**BOH**	**V**	**RS28**	**RS29**	**V**	**BOH**	**RS30**	**CRI**	**BOH**	**RS31**	**RS32**
1	2	18	20	38	18	20	38	38	38	38	38	20	18	38	38	18	20	38	18	20	38	38
**A-genome**																						
A3	I		**2**	2		**2**	2	2	2	2	2	**2**		2	3		**2**	2		**2**	2	2
A1	II		**2**	2		**2**	2	2	2	2	2	**2**		2	1		**2**	2		**2**	2	2
A10	V		**2**	2		**2**	2	2	2	2	2	**2**		2	2		**2**	2		**2**	2	2
A5/6/9*	VIII		**6**	6		**6**	6	6	6	6	6	**6**		4	6		**6**	6		**6**	6	6
**C-genome**																						
C4	IV	**2**		2	**1+1**		2	2`	2`	2	2		**2**	2	2	**2**		2	**1+1**		2	2
C8	VI	**2**		2	**2**		2	2	2	2	2		**2**	2	2	**2**		2	**2**		2	2
C7	VII	**2**		2	**2**		2	2	2	2	2		**2**	2	2	**2**		2	**2**		2	2
**BAC marker*****																						
A-genome (m-)		n.a.	n.a.	19	n.a.	n.a.	20	20	20	20	20	n.a.	n.a.	20	20	n.a.	n.a.	19	n.a.	n.a.	20	20
C-genome (m+)				19			18	18	18	18	18			18	18			19			18	18

S_0 generation: RS23, RS24, RS25, RS26, RS27, RS28, RS29, RS30, RS31 and RS32 lines
S_1 generation: RS5, RS8, RS20, RS21 and RS22 lines
S_2 generation: RS1, RS4, RS6, RS7, RS9, RS10, RS11, RS12, RS13, RS14, RS15, RS16, RS17, RS18 and RS19 lines
S_3 generation: RS2 and RS3 lines

PCH- *B. rapa* ssp. chinensis var. *chinensis (* pak choy)
KO - *B. rapa ssp. rapifera* cv. Kova
SK - *B. rapa ssp. rapifera* cv. Skye
LU - *B. rapa ssp. rapifera* cv. Ludowy
PR - *B. rapa ssp. rapifera* cv. Premium
SA - *B. rapa ssp. rapifera* cv. Salut
BOH - *B. rapa ssp. rapifera* BOH 2877
KI - *B. rapa* ssp. *pekinensis* (chinese cabbage) cv. Kilakin

HG - *B. oleracea* ssp. *acephala* var. *sabellica* cv. Halbhoher Grüner
V- *B. oleracea* ssp. *acephala* var. *sabellica* cv. Vitessa
KA - *B. oleracea* ssp. *acephala* var. *sabellica* cv. Kapral
CRI - *B. oleracea* ssp. *fruticosa* var. *gemmifera* cv. Crispus
